# Epigenetics of Royalty

**DOI:** 10.1371/journal.pbio.1000532

**Published:** 2010-11-02

**Authors:** Alexandra Chittka, Lars Chittka

**Affiliations:** 1 Wolfson Institute for Biomedical Research, University College London, London, United Kingdom; 2 Queen Mary University of London, Research Centre for Psychology, School of Biological and Chemical Sciences, London, United Kingdom

Imagine—you've just been born, and your future looks bleak. After an all-too brief infancy when you'll be cared for and fed, you'll be forced into child labour, cleaning a dark and crowded home and caring for your many siblings. You'll be subsequently put on guard duty to defend your home against vicious intruders. If you survive, you'll spend the rest of your days searching for tiny bits of food from ephemeral sources, mostly not for yourself, but for the communal pantry. Weekends? Holidays? Forget it. Within a few weeks, you'll have worked yourself to death. Moreover, you will never have known love.

Your sister, on the other hand, will begin her career by first murdering her competitors, then sleeping around in grand style. During a string of orgies with thousands of participants, she will fornicate with up to 20 males, who are (literally!) ready to die for the privilege. Upon her return home from such debauchery, she will be treated royally; indeed, for the rest of her life she will be surrounded by loyal staff who will feed, clean her, and cater to her every need. If she should ever have to leave home (which happens rarely) she will be accompanied by thousands of subordinates who will do their best to find a suitable new home. Your sister will live 20 times longer than you and will one day be the proud mother of hundreds of thousands of offspring, while you'll have died a spinster. Unfair? You bet! But then you're just a worker honeybee. As for your sister, it's good to be queen.

## Making Two Fates From One Genome

**Figure 1:**
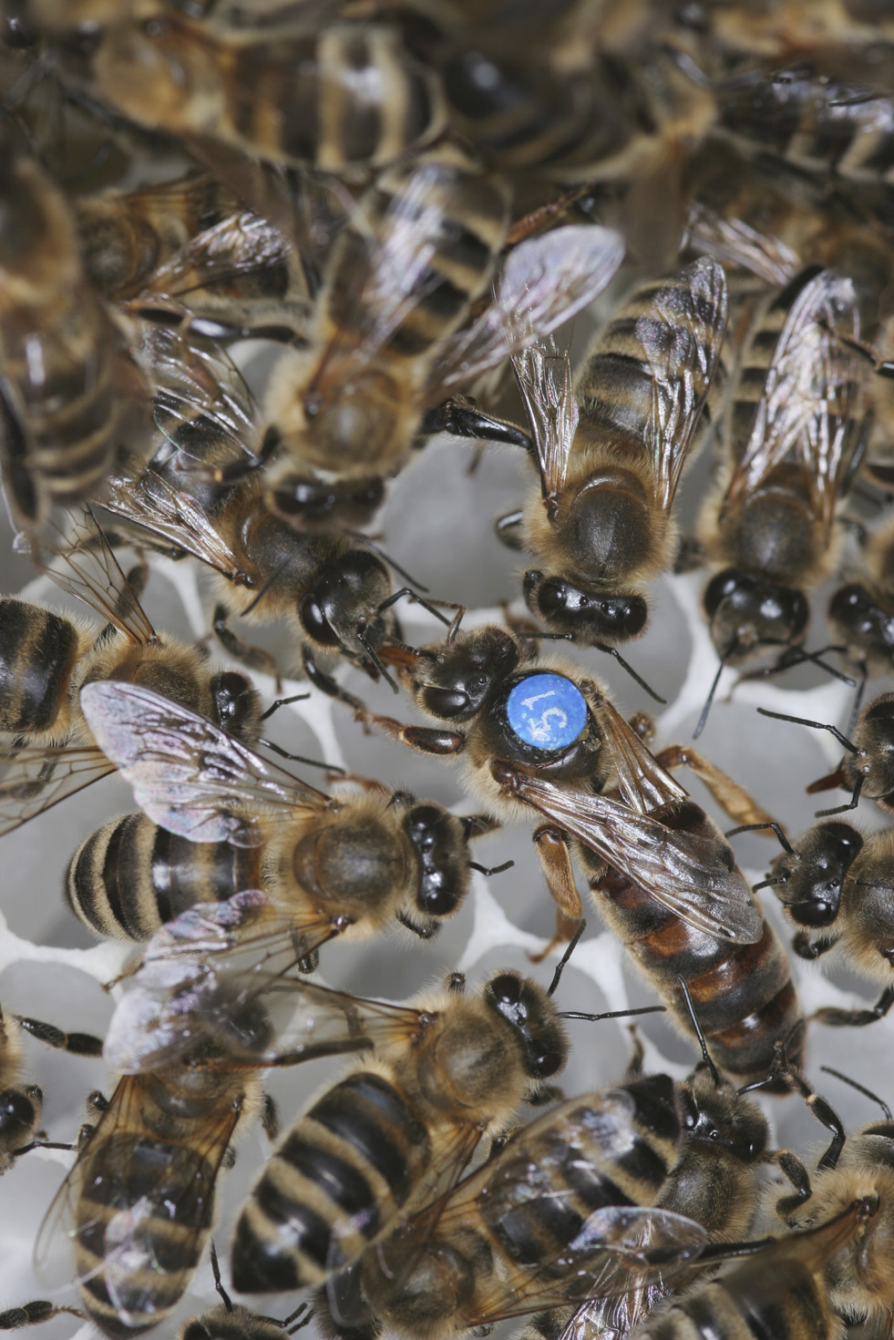
A honeybee queen surrounded by her retinue. There are numerous behavioural, physiological, and anatomical differences between queens (which can lay up to 2,000 eggs per day) and sterile workers, even though they are identical at the genetic level. Upon emergence from the pupae, new queens engage in a series of duels with rival queens. The single survivor will leave the hive for 1–5 mating flights, during which she visits sharply delineated leks—congregation areas used solely for mating that might be several kilometres from the hive, where hundreds of drones typically await. Queens will mate with an average of 12 drones, who die shortly afterwards since the explosive ejaculation ruptures the everted genitals. A mated queen then returns to her native hive; egg laying begins shortly afterwards, and she will typically not leave the colony again unless a new queen is raised in the subsequent year, in which case the old queen leaves the hive with a large swarm of workers to relocate to a new home. Specialised workers who form the queen's retinue feed the queen and constantly groom and lick her, in the process picking up queen mandibular pheromone, which suppresses ovary development in workers [24]. Image: Helga Heilmann, BeeGroup Würzburg.

While royalty is typically heritable in humans and in some social insects [1],[2], this is not so in honeybees. The honeybee queen and workers might be genetically identical but what seals their respective fates is that queen larvae get fed a special diet—royal jelly—in large quantities and over extended periods [3]. This richly nutritious substance's chemical composition is only partially understood and is produced by glands in the mouths of young nurse bees. All larvae are initially fed with royal jelly, although worker larvae are soon weaned and switched to a diet of pollen and nectar, whereas queen larvae are bathed in royal jelly throughout their larval development and feed on it into adulthood. This differential rearing procedure results in striking morphological, behavioural, and physiological differences between these different castes (Figure 1, Table 1). Queens live for years, produce up to 2,000 eggs on a summer day, and never visit flowers (or engage in any activity resembling “work”), while the sterile workers typically live only for weeks, during which they engage in a series of specialisations from cleaning comb cells, tending brood, constructing wax combs, guarding the hive entrance, and, finally, foraging for various commodities such as nectar, pollen, water, and resin [4].

**Table 1:** Overview of differences between queen and workers honeybees.

	Worker	Queen
**Mass at emergence**	81–151mg	178–292mg
**Development egg->adult**	16–24 days	14–17 days
**Age**	15–38 days (summer bees)140 days (winter bees)	1–3 years normally(up to 8 yrs in some cases)
**Facets in compound eye**	5,000–6,000	3,500
**Placoid olfactory sensillae**	2,700	1,600
**Pollen basket**	Yes	No
**Wax glands**	Yes	No
**Spermatheca**	Rudimentary	Large
**Ovarioles**	2–12	150–180
**Sting barbs**	Yes	Rudimentary
**Mandibular glands**	Large	Very large
**Nasonov glands**	Yes	No
**Dance communication**	Yes	No

Note: there is no intention of completeness; there are many more anatomical, neurobiological, and hormonal and behavioural differences between these castes [4],[17],[18],[25].

The observation that different organisms can be generated from identical genomes means that differential gene expression moulds different outcomes from the same genetic material. Honeybees are unique, as different life forms can be entirely induced by diet. The availability of their genome sequence [5] also makes them a unique system to study how environmental stimuli regulate gene expression. Indeed, more than a fifth of the total number of honeybee genes (10,157) are differentially expressed in the brains of queens and workers [5],[6]. A string of recent studies focuses on a particular type of gene regulation—the epigenetic control of gene expression in the remarkable diphenism between queen and worker honeybees [7],[8]. Epigenetics, here, refers to “the structural adaptation of chromosomal regions so as to register, signal, or perpetuate altered activity states” [9], in other words, a departure from the more traditional definition that referred to the heritability of such changes, or their retention through cell divisions.

Changes of chromatin structure affecting transcription are achieved mainly through histone modifications and DNA methylation, where methyl groups are covalently bound to cytosine. This typically happens at CpG-sites, where a cytosine nucleotide occurs next to a guanine nucleotide on the same DNA strand. DNA methylation is traditionally thought to attenuate gene expression [10],[11]. The chromatin structure defined by DNA methylation and histone modifications is reversible and allows for adjustment of transcriptional output to changing environmental conditions or signals.

## Royal Jelly—The Ultimate in Diet Evolution

Since reproductive function is repressed in workers but not queens, it seems possible that DNA methylation results in repression of gene expression in workers. DNA methylation requires the enzyme DNA methyltranferase DNMT3. It was recently shown that silencing DNMT3 expression in newly hatched honeybee larvae mimics the effect of royal jelly, namely, the larvae destined to become workers develop into queens with fully developed ovaries [10]. This was a direct demonstration that royal jelly provides the external information interpreted by the developing larva to create and maintain the epigenetic state necessary to generate a queen. In addition to vitamins, lipids, and amino acids, royal jelly also contains a family of proteins called Major Royal Jelly Proteins, which are thought to be crucial in reproductive maturation [12]. Intriguingly, one of the components of royal jelly is phenyl butyrate, a known histone deacetylase (HDAC) inhibitor [13]. Histone deacetylases catalyse the removal of acetyl groups from histones, which may allow for chromatin to become more compacted, so repressing transcription [14]. These enzymes have been shown to act in concert with DNA methyltransferases [11]. However, workers are not *just* a reproductively repressed form of queens. Workers have a highly differentiated behavioural repertoire, including behavioural routines and cognitive feats which are not displayed by queens [3],[15], and, indeed, many genes are significantly up-regulated in the brains of workers compared to queens [6]. This poses the question of how specificity is generated using epigenetic marks in gene regulation.

## The Honeybee Epigenomes

In this issue of *PLoS Biology*, Lyko et al. report entire methylomes of the brains of honeybee queens and workers in order to obtain a more complete picture of the transcriptional programmes governed by differential DNA methylation [13]. Lyko et al. discovered that most of the DNA methylation in the honeybee genome occurs on CpG dinucleotides in the coding exons of 5,854 genes and only rarely in non-coding, intronic regions (see also [16]). The honeybee genome contains over 10 million CpG sites; only 70,000 cytosines are methylated [13]. In agreement with other studies, methylation of CpGs is common in sections of DNA where CpGs are less ubiquitous, whereas CpG-rich stretches of DNA (so called CpG islands, which are often found in promoter regions of genes) tend not to be methylated [7]. Interestingly, the methylated genes are predominantly not the ones that are specific to *Apis*, but those that are conserved across species, and indeed across phyla [13],[16]. These genes are ubiquitously expressed in many tissues and appear to be involved in basic metabolic processes, whose activities are essential for survival and cannot be switched off entirely. It appears that fine-tuning of the expression levels of these genes in the brain can contribute to dramatic differences in phenotype, although it would be interesting to also explore the methylation patterns in other parts of the body. With respect to reproductive caste differentiation, methylation patterns of over 550 genes differed between queens and workers [13]. Several of these have been shown to be involved in brain development or activity in other species, and, indeed, there are profound differences between the brains of queens and worker honeybees, both in terms of adult anatomy and development [17],[18]. Again, these genes are never “switched off” in either caste. Instead, they are expressed at low to moderate levels, but differentially between castes, strengthening the point that subtle regulation of the metabolic pathways and differentiation genes leading to higher growth rates in queen bees eventually affects reproductive capacity and different behavioural patterns found between the queen and worker bees.

## DNA Methylation and Alternative Splicing

Intriguingly, the authors also found a strong correlation between methylation patterns and splicing sites, including those that can generate alternative exons. This correlation was then investigated in more depth on a selected gene to show that two different splice variants are generated at different levels in queens and workers. Both queens and workers show similar levels of expression of a common long variant of the gene, despite the fact that the gene is heavily methylated in workers but not queens. However, only the queens generate significantly more of the short isoform. The difference in the abundance of the short isoform produced correlates with the differential methylation of CpGs in queens and workers found in the region involved in producing the additional exon in the short isoform; regions near splice sites were conspicuously differently methylated between queens and workers. However, the molecular mechanisms by which this happens are as yet unclear. The observation that the long isoform is produced at comparable levels in both workers and queens despite heavy methylation in the former is matched by recent observations in humans where high levels of gene body methylation can also occur in highly expressed genes [19]. Thus, DNA methylation does not necessarily mediate gene repression, and this raises the question of whether it is not the overall extent of methylation that matters for transcription levels, but instead the particular spatial pattern in the methylation landscape [20], plus various contextual factors [14]. The intriguing observation of non-random distribution of methylated CpGs found near differentially spliced exons in brain DNA underscores the importance of these modifications in generating diversity of gene products from the same template, ultimately leading to functionally different physiological/behavioural outputs [21].

## Conclusion and Open Questions

A key observation that emerges from Lyko et al.'s seminal study [13], and other recent work [22], is that relatively moderate changes in expression of individual genes might act synergistically over many genes to achieve very different physiological, morphological, and behavioural end results. The subtle and sustained nature of input seems to be instrumental in maintaining the cellular activity that is necessary for the production of particular phenotypes. It remains, however, entirely unclear how DNA methyltranferase can “know” which of the ca. 10 million CpGs in the honeybee genome [13] to mark with methyl “tags” [23]. The DNMTs themselves have little specificity for marking particular stretches of DNA and histones. Thus, an intricate interplay between the DNA/histone modifying machinery (the chromatin modifiers) and cellular components with DNA recognition specificity must recruit the chromatin modifying enzymes to the right places at the right times according to signals received by cells. It is also interesting to contemplate why, in some model systems such as *Drosophila*, complex and entirely functional life forms can be generated and maintained largely without the aid of DNA methylation, emphasising that in the absence of some epigenetic modifiers, “traditional” gene regulation can be adapted to perform the functions orphaned by the loss of the phylogenetically ancient epigenetic machinery.

Finally, there is the important question of who decides whether an egg is a queen-to-be or a common worker, and how this decision is made. Workers construct especially large “queen cups” out of wax throughout the season, but they often remain empty. New queens are reared when the old queen dies, or in preparation for colony fission [3]. Eggs are often laid by the queen directly into the cups, in which case she presumably has made a decision that this particular egg should be raised into a queen. However, workers also move eggs from regular worker cells into these queen cups, and queen larvae and pupae are frequently destroyed by workers [4]. Thus, workers can substantially interfere with royal destiny, and this raises the question of whether there is some nepotism involved. While caste differentiation into queens and workers is largely mediated by nutrition in honeybees, there appears to be a genetic influence as well [1]. Because of the promiscuous habits of the queen, a honeybee hive typically contains multiple subfamilies of workers each fathered by a different drone, and some subfamilies can be substantially overrepresented in queen production, presumably mediated by preferential treatment of certain larvae and selective abortion of others. This adds a level of complexity to the interplay between genomic and environmental factors: the provisioning of epigenetic factors via larval nutrition might in turn be controlled by genetic factors that control provisioning behaviour.
